# The protective impact of growth hormone against rotenone-induced apoptotic cell death via acting on endoplasmic reticulum stress and autophagy axis

**DOI:** 10.55730/1300-0152.2639

**Published:** 2022-12-15

**Authors:** Özge BERRAK RENCÜZOĞULLARI, Selay TORNACI, Yağmur ÇELİK, Nayat Narot CİROĞLU, Pınar OBAKAN YERLİKAYA, Elif Damla ARISAN, Ajda ÇOKER GÜRKAN

**Affiliations:** 1 Department of Molecular Biology and Genetics, İstanbul Kültür University, İstanbul, Turkey; 2 Department of Molecular Biology and Genetics, Medeniyet University, İstanbul, Turkey; 3 Institute of Biotechnology, Gebze Technical University, Gebze, Turkey; 4 Department of General Biology, Faculty of Science, Marmara University, İstanbul, Turkey

**Keywords:** Rotenone, Parkinson’s disease, growth hormone, autophagy, endoplasmic reticulum stress

## Abstract

Human growth hormone (GH) is crucial modulator of cellular metabolisms, including cell proliferation and organ development, by stimulating insulin-like growth factor-1 (IGF-1), which has various functions such as cell proliferation, tissue growth, survival, or neuroprotection. Therefore, GH is implicated as a critical player in the cell and can enhance neurogenesis and provide neuroprotection during the treatment of neurological diseases such as Parkinson’s disease (PD). In this study, the neuroprotective role of GH was investigated in rotenone-induced PD models for the first time. Both SH-SY5Y and SK-N-AS neuroblastoma cells were exposed to rotenone to mimic PD pathogenesis as stated in previous studies. Our data demonstrated that overexpression of GH led to the resistance of the SH-SY5Y and SK-N-AS cell lines to rotenone treatment. The levels of ER stress markers, CHOP, PERK, XBP-1, and ATF6, were higher in wt cells than GH+ SH-SY5Y cells. However, the level of autophagy markers LC3 increased and the levels of reactive oxygen species (ROS) decreased with the overexpression of GH. Furthermore, while rotenone significantly increased the SubG1 population in the cell cycle of SH-SY5Y wt cells, there was a minor alteration in GH+ cell population. Concomitantly, the levels of the proapoptotic marker, cleaved-PARP, and positive staining of Annexin V in SH-SY5Y wt cells were higher after rotenone treatment. Together, these results revealed that overexpression of GH enhanced the autophagy response by triggering the ER stress of SH-SY5Y cells to rotenone exposure and showed a neuroprotective effect in vitro PD models.

## 1. Introduction

Parkinson’s disease (PD), the second most common neurological disorder, is characterized by the loss of dopaminergic neurons in the substantia nigra region of the cerebrum (Forno, 1988). The exact molecular mechanism underlying the deterioration of neuronal cells has not been elucidated yet. Mitochondrial dysfunction, exposure to neurotoxins, and genetic factors such as Parkin and leucine-rich repeat kinase 2 (LRRK2) were assumed to be associated with PD pathogenesis (Fukae et al., 2007). Although so many decades of in vivo, in vitro, and epidemiological studies were performed, new pharmacotherapeutic approaches are still needed to protect the dopaminergic neuronal cell death. L-dopa, selegiline (MAO-B inhibitor), and Amantadine are given with L-dopa alone or in combination, especially during the late stage of PD, are promising innovative agents to have the potential to slow or reduce the PD progression (DeMaagd and Philip, 2015). Clinical studies also indicate that pesticides cause neurotoxic effects and compose a risk factor for PD.

Rotenone, a natural insecticide extracted from *Derris elliptica* and *Lonchocarpus utilie*, has an impact on dopaminergic neuron loss (Park and Kim, 2013). Rotenone treatment was shown to trigger apoptotic cell death due to loss of mitochondria membrane potential of dopaminergic neurons. Caspase-3 dependent apoptotic cell death was exhibited following rotenone exposure in HL-60 and ventral mesencephalic dopaminergic neurons (Lin et al., 2012). In SH-SY5Y cells, rotenone triggered c-Jun N-terminal protein kinase-mediated stress activation was reported. In contrast, the activation of caspase-3 and JNK have not been displayed in rotenone-treated PC12 cells (Newhouse et al., 2004). The systemic exposure of rotenone led to the dysfunction of Parkinsons’-related gene expressions (α-synuclein, parkin) in neuroblastoma cells which made the cells a valuable rotenone model of Parkinsons’ disease. It was reported that rotenone induced oxidative damage might be due to accumulation of α-synuclein or reduction of complex I (Drolet et al., 2009). Rotenone administration to male Lewis rats generated in vivo PD models through the dopaminergic neuronal loss of *Substantia nigra*. Thus, rotenone was used as an efficient agent to generate PD in vitro and in vivo models for studying the molecular machinery of neuroprotection (Ma et al., 2018). Recent epidemiological studies demonstrated the neuroprotective role of insulin-like growth factor-I (IGF-I) against stress signals from lymphocytes of PD patients (Lewitt and Boyd, 2019). Besides, the expression of IGF-I receptors in lymphocytes supports the potential neuroprotective effect of IGF-I (Lewitt and Boyd, 2019). Moreover, IGF-I interferes with rotenone-mediated apoptotic cell death of lymphocytes via inducing the PI3K/Akt pathway, inhibiting p53 expression (Duan and Maki, 2016). Also, growth hormone releasing peptide-6 (GHRP-6) neuroprotective effect has been displayed in adult male rats’ cerebellum, hypothalamus, and hippocampal neuronal cells via increased phosphorylation of PI3K/Akt and Bad through IGF-I upregulation (Duan and Maki, 2016). The age-related cerebral cell death interferes through caspase-3 and -9 activation following GHRP-6 mediated IGF-I expression (Pañeda et al., 2003). GH, an essential pituitary-derived hormone necessary for postnatal human development via acting on muscle, bone, and fat tissue. The biological activation of GH is under the control of IGF-I expression following its binding on GHR on target tissues (Duan and Maki, 2016).

Rotenone induced endoplasmic reticulum (ER) stress in SH-SY5Y and SK-N-AS neuroblastoma cells (Karmakar et al., 2011). Many neurological disorders such as Alzheimer’s disease, amyotrophic lateral sclerosis, and PD occurred because of ER stress from the accumulation of the misfolded proteins, dysregulation of extracellular/intracellular signaling, which controls calcium secretion (Montibeller and De Belleroche, 2018). Unfolded protein response (UPR) is activated by the accumulation of the misfolded proteins in the ER lumen (Adams et al., 2019). Autophagy is a catabolic process where damaged organelles or disrupted proteins are absorbed and degraded for recycling to prove cellular homeostasis (Kaur and Debnath, 2015). The UPR and autophagy mechanisms work simultaneously and are associated with the regulation of pathological processes, such as tumorigenesis and neurodegeneration. There are three pathways related to ER stress and autophagy: (1) PERK/eIF2α/ATF4/CHOP signaling, (2) IRE1α/JNK1/XBP-1 signaling, and (3) ATF6 signaling pathways (Chipurupalli et al., 2021). Autophagy has contributed to suppressing various neurodegenerative diseases. Parkin and PINK1 are associated with familial PD, which can initiate the autophagy process to eliminate the damaged mitochondria. Furthermore, the accumulation of PINK1 leads to ubiquitination of damaged mitochondria by Parkin, a ubiquitin ligase, and causes accumulation of p62, a key marker of autophagy (Deas et al., 2011).

Furthermore, in vivo studies of various therapeutic agents such as celastrol or rapamycin led to the activation of autophagy and suppressed the accumulation of α-synuclein PD models (Deng et al., 2013). GH also has a neuroprotective role and can enhance neurogenesis by activating its downstream targets, including the JAK-STAT, MAPK, or PI3K pathways, which play critical roles in neuronal proliferation (Chung et al., 2015). Several evidence showed that JAK-2 dependent GH pathways stimulate ERK phosphorylation for neuronal proliferation (Devesa et al., 2014). Although the increase secretion of GH in PD patients were known, the role of ER-stress and autophagy axis in GH overexpressing PD in vitro models has not been investigated yet. This study aims to evaluate the potential protective effect of growth hormone against rotenone-induced apoptotic cell death in SH-SY5Y and SK-N-AS neuroblastoma cell lines through acting on endoplasmic reticulum stress and autophagy mechanisms.

## 2. Material and methods

### 2.1. Cell culture, chemicals, and antibodies

SH-SY5Y (CRL-2266) and SK-N-AS (CRL-2137) human neuroblastoma cell lines were purchased from American Type Culture Collection (ATCC, Rockville, MD, USA). Cells cultured in DMEM (PAN-Biotech GmbH, Aidenbach, Germany) medium supplemented with 10% fetal bovine serum (PAN-Biotech GmbH) and 1% penicillin/streptomycin (GIBCO, Invitrogen) in a humidified atmosphere containing 5% CO_2_ at 37 °C incubators (Hera Cell 150i, Thermo Lab systems, Beverly, MA, USA). Forced GH was expressing SH-SY5Y cells generated according to our previous work (Coker-Gurkan et al., 2018). Mycoplasma testing has been done for the cell lines used by PlasmoTest Reagent Kit (InvivoGen, Toulouse, France). Rotenone (Sigma, St. Louis, MO, USA) was dissolved in dimethyl sulfoxide (DMSO; 10 mM). The following antibodies; BIP, CHOP, PERK, p-PERK (Thr 980), Ire1α, p-Ire1α (Ser724), ATF6, XBP-1, eIF2α, SAPK/JNK, Beclin-1, Atg12, Atg 5, Atg 16, LC3, p62, PARP, caspase-7, Bim, Bax, Puma, Bcl-2, and polyclonal antirabbit/mouse antibodies were purchased from Cell Signaling Technology (CST, Danvers, MA, USA). Each primary antibody was diluted to 1:1000 and HRP-conjugated secondary antibodies were diluted to 1:3000 with in superblock T20 reagent from Thermo Scientific (Beverly, MA, USA).

#### RNA extraction and RT-PCR

Trizol RNA extraction buffer was used to isolate RNA isolation of SH-SY5Y wt and SH-SY5Y GH+ cells. According to the manufacturer’s instructions, RNA reverse-transcribed to cDNA using the iScript cDNA Synthesis Kit (Quantabio, Beverly, MA, USA). Then, 1000 ng of cDNA was used for PCR reaction. Expression of GH (Gene ID: 2688) was determined by forward and reverse primers against the GH gene; 5′ AGGATCCCAAGGCCCC-3′,5′-TAGAAGCCACAGCTGCCCTCCAC -3′, respectively. Expression of 18S (Gene ID: 6222) was determined by forward and reverse primers against the 18S gene 5′-GCAGAATCCACGCCAGTACAAG-3′ and 5′-GCTTGTTGTCCAGACCATTGGC-3′, respectively.

### 2.2. GH ELISA

Secreted GH levels were determined in a filtered medium of 24 h serum-starved SH-SY5Y wt and GH+ cells by human GH ELISA Assay (Abcam, 190811) according to the manufacturer’s instructions.

### 2.3. MTT cell viability and cell growth assay

MTT cell viability assay was performed to understand the effect of rotenone in SH-SY5Y wt and GH+ cells. For this purpose, 1 × 10^4^ cells/well seeded into 96-well plates and treated with rotenone (0.1, 0.5, 1, 5, and 10 μM) for 24 h. Ten microliters μL MTT reagent added until 4 h incubation at 37 °C. Two hundred microliters DMSO were applied, and cells were incubated in the dark for 5 min. Then, an ELISA reader was used for the quantification at 570–655 nm. Trypan blue assay was performed to determine the effect of 0.5 μM otenone in a time-dependent manner (24, 48, and 72 h) on cell proliferation. Then, 5 × 10^4^ cells/well seeded into 6-well plates. 0.4 % w/v Trypan blue was used at 1:1 to distinguish between the viable and dead cells. Then viable cells were counted with EVE automatic cell counter (NanoEnTek, Cambridge, UK).

### 2.4. Fluorescence microscopy

Firstly, 1 × 10^5^ cells seeded in 6-well plates and treated with rotenone at 0.1, 0.5, 1, 5, and 10 μM for 24 h. Mitochondrial membrane potential (MMP) determined by 3,3′-Dihexyloxacarbocyanine iodide (DiOC_6_) staining. PBS washed cells stained with DiOC6 (0.4 mM) and visualized by fluorescence microscopy (Olympus IX70, Tokyo, Japan) at Ex. /Em. = 485/538 nm. Cell death was determined the following propidium iodide (PI) staining (Ex./Em.:493/636 nm). 4′,6-diamidino-2-phenylindole (DAPI) was used to observe rotenone-induce DNA double-stranded breaks (Ex./Em.: 358/461 nm). Acridine orange was used to determine the vacuole formations in different pH following rotenone treatment. The starvation protocol performed as a positive control (Ex./Em.: 460/650 nm). mCherry-CHOP plasmid-transfected cells were visualized under the microscope following 3-, 6-, 9-, and 24-h treatment of 0.5 μM rotenone. One millimolar tunicamycin was used as a positive control (Ex./Em.:550/650 nm). Images were taken at different time points and analyzed using the Olympus Micro DP Manager Image Analysis program and presented by graphics.

### 2.6. Soft agar colony formation assay

The bottom layer of the 6-well plate was covered by 0.5 % agarose and 2× DMEM (1:1). Then the upper layer with 0.3% agarose solution, 5 × 10^4^ cells in 2× medium was placed on. Let the cells to form colonies for 21 days at 37 °C, 5% CO_2_. Analysis of spheroid forms by number and diameter was performed by Olympus Micro DP Manager Image Analysis. Colonies stained with DiOC6 and DAPI after 21 days.

### 2.7. Flow cytometry

mCherry-CHOP analysis: 5 × 10^5^ cells seeded into 6-well plates and transfected with the mCherry-CHOP-contained plasmid with the Fugene HD transfection reagent and incubated for 48 h. Then, cells were treated with 0.5 μM rotenone for 3, 6, 9, and 24 h. One millimolar tunicamycin was used as a positive control. Then, 1 × 10^4^ cells were analyzed by FL3 filter of BD Accuri C6 flow cytometer (BD Bioscience, San Jose, CA, USA) and normalized with the untreated SH-SY5Y wt cells. The percentages of mCherry-CHOP contained cells were determined with the graphic.

GFP-LC3 analysis: Cells were transfected with GFP-LC3-contained plasmid as described previously. Then cells were treated with 0.5 μM rotenone for 24 h. Later, cells were analyzed by the FL1 filter of the BD Accuri C6 flow cytometer and presented with the graphic.

DCFH-DA staining analysis: Cells treated with 0.5 μM rotenone in the presence and absence of N-acetylcysteine (NAC) for 24 h. Cells were stained with 5 μM DCFH-DA (Molecular probes, Oregon, USA) for 15 min at 37 °C, 5% CO_2_. Then stained cells analyzed with flow cytometer.

Cell cycle analysis: Cells treated with 0.5 μM rotenone in the presence and absence of NAC for 24 h. Cell cycle analysis has proceeded as described in our previous study (Rencuzogulları et al., 2020). Briefly, cells were fixed with cold 70% ethanol for two days. After fixation, cells were centrifuged at 300 g. Cell pellets were washed with PBS. Then, cells were stained with 1 μg/mL PI and 5 μg/mL RNase for 30 min at dark. FL2 filter of the BD Accuri C6 flow cytometer was used to analyze the cell cycle phases.

Annexin V and PI staining: Following 0.5 μM rotenone treatment for 24 h, cells were trypsinized. Then cells stained with FITC-conjugated Annexin V and propidium iodide (PI) analyzed by using a flow cytometer to examine the apoptotic cells.

### 2.8. Western blotting

M-PER Mammalian Protein Extraction Reagent (Thermo Scientific, USA) was used for total protein extraction as described in our previous study (Coker-Gurkan et al., 2018). Bradford protein assay was performed for determining the total protein concentrations of SH-SY5Y wt and GH+ cells (Bio-Rad, USA). Then, 30–50 μg protein was separated on 10%–15% sodium dodecyl sulfate-polyacrylamide gels (SDS-PAGE) according to protein molecular weight and transferred polyvinylidene difluoride (PVDF) membranes (Thermo Scientific, USA). Then PVDF membranes incubated in the primary antibody (overnight at 4 °C) at 1:1000 concentration (BIP, CHOP, PERK, Ire1α, ATF6, XBP-1, eIF2α, SAPK/JNK, Beclin-1, Atg12, Atg 5, Atg 16, LC3, p62, PARP, caspase-7, Bim, Bax, Puma, Bcl-2, β-actin) and then with secondary antibodies (antirabbit IgG or antimouse IgG) overnight at 4 °C. Homemade chemiluminescence reagent (31) were used to develop the membranes. All proteins were normalized to the loading control β-actin.

### 2.9. Statistical analysis

All the statistical analyses of the experiments were performed using GraphPad Prism version 8 (https://www.graphpad.com/). Immunoblotting images were analyzed by using ImageJ (https://imagej.net/Welcome). Two-way ANOVA and Sidak’s or Tukey’s multiple comparison test was used for analyzing the statistics of all experiments which were repeated at least three times. Error bars represent the average ± standard deviation values. Significance levels represented as the following: * p < 0.05; ** p < 0.01; *** p < 0.001; **** p < 0.0001.

## 3. Results

### 3.1. Protective effect of GH expression against rotenone-induced cell death in SH-SY5Y and SK-N-AS cells

**Figure 1:**
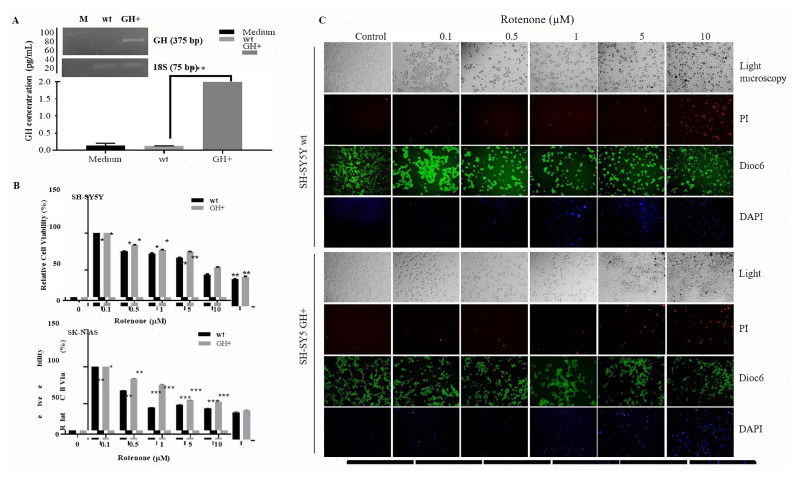

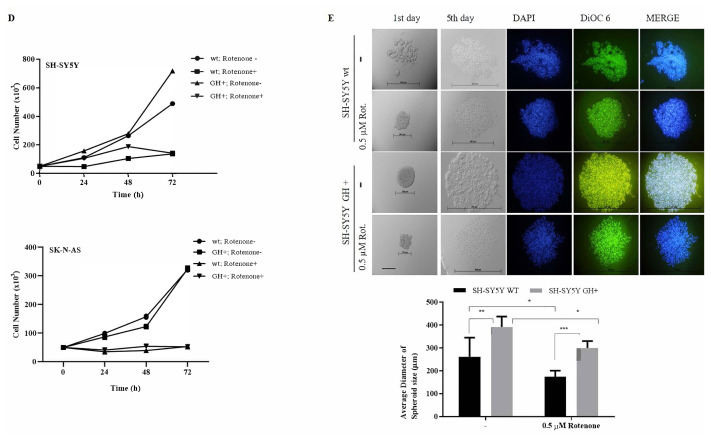

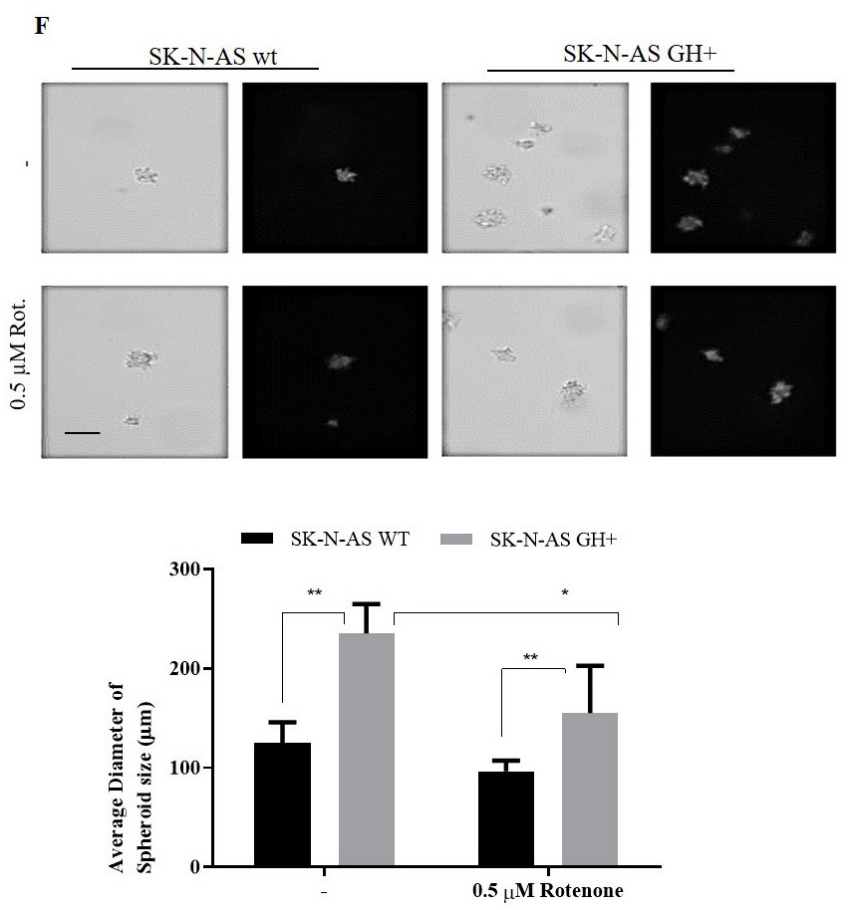
Rotenone treatment reduced cell viability in SH-SY5Y wt cells more than in human autocrine GH-gained SH-SY5Y cells. A. GH secretion of SH-SY5Y following GH-pcDNA3.1 plasmid transfection was determined as 1.98 pg/mL by ELISA assay. B. MTT cell viability assay was performed in rotenone treated-SH-SY5Y and SK-N-AS cells with/without GH in a dose-dependent manner (0.1, 0.5, 1, 5, and 10 μM). * p < 0.05, **p < 0.01, ***p < 0.001. C. The effect of rotenone on MMP loss-induced cell death was determined by DiOC6, PI, and DAPI staining in both SH-SY5Y wt and GH+ cells. D. Cell survival assay was performed to show the effect of rotenone in a time-dependent manner in both SH-SY5Y and SK-N-AS wt (upper figure) and GH+ (lower figure) cells. E–F. The effect of 0.5 μM rotenone on anchorage-independent cell growth was examined by soft agar assay in SH-SY5Y (magnification: 40×) and SK-N-AS (magnification: 10×) cell lines. Scale bar: 200 μm. Average diameter of spheroids for both Figures E and F was analyzed by Olympus Image Analyzing program.

To understand the effect of GH on cell proliferation and drug-resistant mechanisms in neuroblastoma, both SH-SY5Y and SK-N-AS cells transfected with GH gene inserted plasmid (Figure 1A). Following PCR analysis, the mRNA expression level of GH was detected by a 2-fold increase in SH-SY5Y cells compared to control cells. The effect of rotenone on cell viability was determined by MTT cell viability assay in a dose-dependent manner (0.1, 0.5, 1, 5, and 10 μM) for 24 h in each neuroblastoma cell with/without GH (Figure 1B). Cell viability was decreased in a dose-dependent manner of rotenone which 0.5 μM concentration led to a decrease of cell viability by 25% and 20% SH-SY5Y and SH-SY5Y+GH cells, respectively (* p < 0.05). SK-N-AS cells were more sensitive to rotenone treatment, which induced remarkable downregulation of cell viability even in 0.1 μM treatment. At a higher concentration of rotenone (0.5, 1, 5, and 10 μM) the cell viability was around 40% in SK-N-AS cells (* p < 0.01). However, GH+ SK-N-AS cells were more resistant to rotenone treatment than wt cells. Additionally, SH-SY5Y cells stained with DiOC6, PI, and DAPI following drug treatment in indicated concentrations, then visualized by fluorescence microscopy (Figure 1C). Rotenone induced MMP loss in a dose-dependent manner in SH-SY5Y cells, albeit this effect was less in GH+ cells. Concomitantly, PI-stained cells were more observable in a higher concentration of rotenone for each cell line. It also observed that rotenone increased DNA damage. However, it was clear that GH+ cells were more resistant to rotenone compared to wt cells. Then, 0.5 μM rotenone, as a neurotoxic drug candidate, was selected for further experiments to form a Parkinson’s disease model. The cell proliferation ability of cells inhibited by treatment in a time-dependent manner. A significant difference was found between wt and GH+ rotenone treated neuroblastoma cells within 48 h. However, no statistical significance was determined following 72 h rotenone exposure between wt and GH+ cells (Figure 1D). It observed that the proliferation rate of GH+ cells was higher than wt cells in both SH-SY5Y and SK-N-AS cells. Record of 490 × 10^3^ (±5) cells detected for untreated SH-SY5Y cell at 96 h was brought down to 136 × 10^3^ (±5) cells following 0.5 μM rotenone treatment. Although the cell proliferation rate was suppressed by rotenone, the cytostatic effect of rotenone continued for 24–72 h in SH-SY5Y GH+ cells. The rate of cell proliferation for SH-SY5Y GH+ cells in response to the following 0.5 μM rotenone was reduced from 720 × 10^3^ to 140 × 10^3^ cells. The metastatic potential of SH-SY5Y and SK-N-AS cells was illustrated by soft agar assay (Figures 1E and 1F). It observed that GH+ cells showed extensive growth with the anchorage-independent for five days. However, rotenone treatment significantly reduced the anchorage-independent growth of both SH-SY5Y wt and GH+ cells. The number and diameter of colonies were higher in GH+ cells than SK-N-AS wt cells. While rotenone treatment did not significantly affect the diameter of SK-N-AS wt spheroids, GH+ SK-N-AS spheroid numbers and the diameters of spheroids were reduced by rotenone treatment significantly (Figure 1F).

### 3.2. GH prevented elevation of ER stress during Rotenone treatment in SH-SY5Y cells

**Figure 2:**
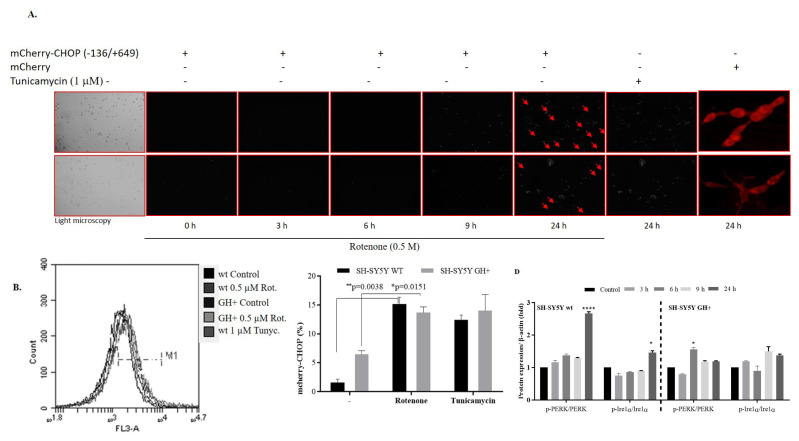
ER stress was induced by rotenone treatment in SH-SY5Y wt cells. A. mCherry-CHOP-transfected cells were treated with rotenone in a time-dependent manner and visualized by fluorescence microscopy to observe the CHOP mediated-induced ER stress in SH-SY5Y wt and GH+ cells. B. Rotenone-treated SH-SY5Y wt and GH+ following mCherry-CHOP transfected cells were determined by flow cytometric analysis. Tunicamycin was used as a positive control for ER stress * p < 0.05, ** p < 0.01 C. The expression levels of BIP, CHOP, PERK, Ire1α, ATF6, XBP-1, eIF2α, and SAPK/JNK were examined by Western blotting in rotenone-treated SH-SY5Y wt and GH+ cells in a time-dependent manner. D. The levels of pPERK/PERK and pIREα/IREα were evaluated in SH-SY5Y wt and GH+ cells. β-actin was used to normalize the expression levels of each protein. *p < 0.05, **p < 0.01, ***p < 0.001 and ****p < 0.0001.

The effect of ER stress on the inhibition of cell proliferation was determined following rotenone treatment by transfection of the mCherry-CHOP plasmid to SH-SY5Y wt and GH cells (Figure 2A). It was observed by fluorescence microscopy that 24-h treatment of rotenone caused a significant increase in the CHOP expression in SH-SY5Y wt and GH+ cells. However, rotenone-induced CHOP expression was less in GH+ cells when compared with SH-SY5Y wt. An ER stress inducer, tunicamycin, was used as a positive control to compare the expression level of CHOP following rotenone treatment in each cell line (Figur 2A). The mCherry-CHOP plasmid transfected cells were analyzed by flow cytometry following 0.5 μM rotenone treatment for 24 h. Consistently with fluorescence microscopy results, there was a further increase in CHOP expression in wt cells than GH+ cells when each compared with their untreated cells (Figure 2B). The expression levels of BIP, IRE1α, p-IRE1α, PERK, p-PERK, CHOP, eIF2α, ATF 6, and XBP1 were displayed by Western blotting in SH-SY5Y wt and GH+ cells (Figure 2C). Levels of BIP, PERK, IRE1α, ATF 6 in SH-SY5Y wt are higher in the early time points (3h, 6h, and 9h) after treatment with rotenone compared with GH+ cells.

Moreover, a significant increase in the expression levels of PERK, CHOP detected in 24-h treatment of rotenone in wt cells and GH+ cells. In addition, the p-PERK levels significantly increased by 24-h rotenone treatment in both wt and GH+ SH-SY5Y cells, but there was no significant change at early time treatment of rotenone. Additionally, forced expression of GH reduced rotenone-induced XBP-1 levels in the late time point of treatment compared to SH-SY5Y wt cells. It also detected that JNK expression levels, which were related to the ER-stress-induced apoptosis, were increased by rotenone treatment in SH-SY5Y wt cells, while there was no significant increase in GH+ cells. Evaluation the levels of pPERK/PERK and pIREα/IREα showed that 24-h rotenone treatment significantly increased the ER stress related signaling through activation of PERK and IREα, but overexpression of GH led to prevention of ER stress in SH-SY5Y cells (Figure 2D). Therefore, it could be concluded that GH had a protective effect on rotenone-induced ER stress in SH-SY5Y cells.

### 3.3. Forced expression of GH positively regulated autophagy during rotenone treatment

**Figure 3:**
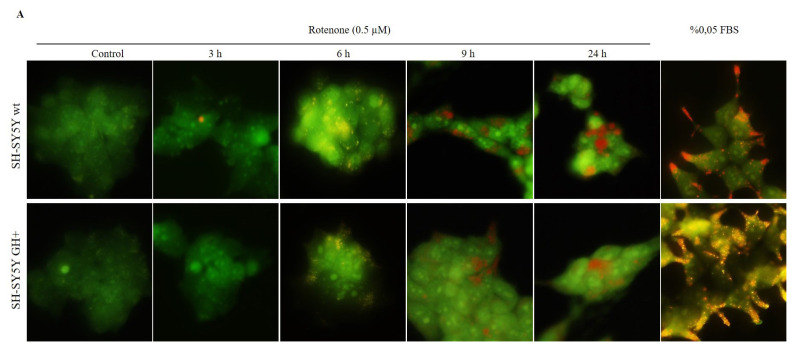

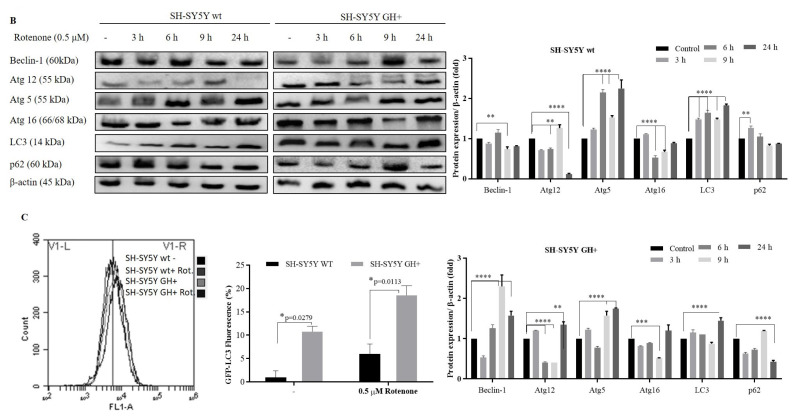
GH positively regulated autophagy following rotenone treatment in SH-SY5Y cells. A. Acridine orange staining was performed to observe the vacuole formation in different pH following rotenone treatment in a time-dependent manner in SH-SY5Y wt and GH+ cells. Starvation with 0.05 % FBS was used as a positive control. B. The expression levels of Beclin-1, Atg12, Atg5, Atg16, LC3, and p62 were determined by Western blotting in a time-dependent manner treatment of rotenone in both SH-SY5Y wt and GH+ cells. The expression levels were normalized to β-actin levels of each protein. **p < 0.01, ***p < 0.001, ****p < 0.0001 C. GFP-LC3 transfected cells were treated with 0.5 μM rotenone, and the induction of autophagy was analyzed by flow cytometry for each cell line.

To elucidate the protective role of GH for decreasing ER stress in SH-SY5Y cells, the autophagy mechanism was evaluated. Firstly, the acridine orange staining was performed following rotenone treatment in a time-dependent manner. There was no significant change in the number of acidic vacuoles until 9-h treatment of rotenone in SH-SY5Y wt and GH+ cells. Starvation of cells by incubation with 0.05% FBS for 2 h was used as a positive control for each cell line. To further understand if these vacuoles were associated with autophagy, the expression levels of Beclin-1, Atg12, Atg5, Atg16, p62, and LC3 were determined. The phagophore initiation factor Beclin-1 levels were higher at early time rotenone treatment compared to the 24-h treatment in both SH-SY5Y wt and GH+ cells. The levels of the autophagosome complex members Atg12, Atg5, and Atg16, increased in a time-dependent manner in each cell line. Also, the late autophagosome formation member p62 levels decreased in a time-dependent manner of rotenone treatment in each cell line. The lapidated form of LC3 which is 14 kDa, LC3IIB, a specific marker of autophagy, was significantly increased by rotenone treatment in each cell line. However, all results obtained from immunoblotting showed that GH+ cells had more vulnerability to rotenone treatment to induce autophagy in SH-SY5Y wt cells. We further investigated the rotenone-induced autophagy by transfection of GFP-LC3 to SH-SY5Y wt and GH+ cells. Compared to untreated SH-SY5Y wt cells, rotenone further increased the levels of GFP-LC3 in SH-SY5Y GH+ cells than wt cells (Figure 3C). These data suggested that the 24-h treatment of rotenone upregulated the autophagy mechanism in SH-SY5Y GH+ cells.

### 3.4. GH prevented rotenone-mediated apoptotic cell death in SH-SY5Y cells

**Figure 4:**
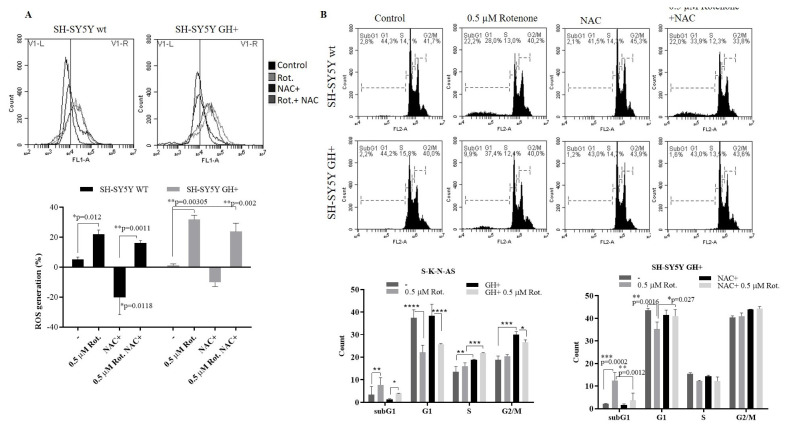
GH had a neuroprotective role during rotenone treatment in SH-SY5Y cells. **A.** The role of ROS generation in rotenone-induced cell death in SH-SY5Y wt and GH+ cells was determined by flow cytometry following DCFH-DA staining of cells. Cells were treated with 0.5 μM rotenone with/without 5 μM NAC. SH-SY5Y wt: *p = 0.012 control vs. 0.5 μM rotenone, *p = 0.0135 Control vs. NAC+, **p = 0.0011 NAC+ vs. NAC+0.5 μM rotenone; SH-SY5Y GH+ cells: **p = 0.003 Control vs. 0.5 μM rotenone, **p = 0.002 Control vs. NAC+0.5 μM rotenone. B. Cell cycle analysis was performed by flow cytometry following PI staining in 0.5 μM rotenone-treated cells in the presence and absence of NAC. SH-SY5Y wt SubG1: ****p < 0.0001 Control vs. 0.5 μM rotenone, G1: ****p < 0.0001 Control vs. 0.5 μM rotenone, 0.5 μM rotenone vs. NAC+ 0.5 μM rotenone, G2/M: ****p < 0.0001 0.5 μM rotenone vs. NAC+ 0.5 μM rotenone. SH-SY5Y GH+ G1: ***p = 0.0002 Control vs. 0.5 μM rotenone, **p = 0.0012 0.5 μM rotenone vs. NAC+ 0.5 μM rotenone, G1: **p < 0.0016 Control vs. 0.5 μM rotenone, *p = 0.27 0.5 μM rotenone vs. NAC+ 0.5 μM rotenone. Ns = nonsignificant. C. Cell cycle analysis of SK-N-AS wt and GH+ cells following rotenone treatment * p < 0.05, ** p < 0.02, ****p < 0.0001. D. The expression levels of caspase-9, caspase-7, Bax, and Bcl-2 were examined by Western blotting following 0.5 μM rotenone treatment of SK-N-AS wt and GH+ cells. E. The expression levels of PARP, caspase-7, Bim, Bax, Puma and Bcl-2 were examined by Western blotting following 0.5 μM rotenone treatment of SH-SY5Y wt and GH+ cells. The expression levels were normalized to β-actin levels of each protein. ***p < 0.001, ****p < 0.0001. F. The apoptotic ratio of cells following 0.5 μM rotenone treatment was analyzed by flow cytometry following Annexin V/PI staining **p = 0.0013 ***p = 0.007, ****p < 0.0001.

To clarify, the effect of rotenone on decreased cell viability was attributable to apoptosis. Firstly, we performed MitoTracker Red staining following 0.5 μM rotenone treatment for 24 h. Rotenone induced MMP loss in each cell line (Figure S1). To evaluate the level of reactive oxygen species (ROS), cells were stained with DCFH-DA and analyzed by flow cytometry following rotenone treatment of cells. Three-hour treatment of rotenone led to a significant increase of ROS generation in both SH-SY5Y wt and GH+ cells. The ROS generation in the 3-h treatment with rotenone reached the highest value in GH+ cells. Six-, 9-, and 24-h treatment with rotenone also showed increased-ROS levels compared to untreated SH-SY5Y GH+ cells (Figure S2). However, cotreatment of NAC with rotenone prevented the rotenone-induced ROS generation more significantly in SH-SY5Y GH+ cells than wt cells. To understand the effect of ROS generation on the cell cycle, flow cytometric analysis of PI staining was performed following cotreatment of NAC with rotenone in each cell line (Figure 4A). Consistently with cell viability assays, rotenone treatment significantly increased the SubG1 levels by 22.5% and 9% in SH-SY5Y wt and GH+ cells, respectively. Although attenuation of ROS reduced the SubG1 levels in GH+ cells, there was no change in SH-SY5Y wt cells following rotenone treatment. Therefore, it be concluded that the increase of the SubG1 population was independent of ROS generation in SH-SY5Y wt cells. However, the SubG1 population was significantly reduced by NAC cotreatment in SH-SY5Y GH+ cells (Figure 4B). Thus, the increase of the apoptotic population might be in a ROS-dependent manner in GH+ cells. GH led to increasing in S and G2/M population rates of SK-N-AS cells. The rotenone-induced subG1 levels were higher in wt cells than GH+ SK-N-AS cells. Moreover, the GH+ SK-N-AS cells arrested at the G2/M phase after rotenone treatment. We further performed Western blot analysis of pro- and antiapoptotic members after 24-h treatment of rotenone. Rotenone treatment decreased the procaspase-7 and caspase-9 expressions in SK-N-AS wt cells. Whereas full caspase-9 levels increased in GH+ SK-N-AS (Figure 4D). However, Bax levels were also downregulated by rotenone in wt and GH+ SK-N-AS cells. While antiapoptotic Bcl-2 levels decreased in wt SK-N-AS cells, there was the opposite effect in GH+ SK-N-AS cells. The significant increase of cleavage of PARP in wt cells was attenuated by forced GH+ expression following rotenone treatment. Rotenone decreased the total caspase-7 in SH-SY5Y wt cells while there was no decrease in SH-SY5Y GH+ cells. BIM, an important essential mediator of apoptosis signaling cleavage, an important essential mediator of apoptosis signaling, was further triggered by rotenone treatment in SH-SY5Y than GH+ cells. The expression levels of proapoptotic members Bax and PUMA recorded 2-fold increase in SH-SY5Y wt cells compared to GH+ cells. Antiapoptotic Bcl-2 levels decreased by rotenone treatment in each cell line (Figure 4E). To detect the effects of GH expression on rotenone-induced apoptosis, we performed the Annexin V/PI staining (Figure 4F). Consistent with immunoblotting results, increased-GH expression reduced apoptotic cell death in rotenone-treated SH-SY5Y cells.

## 4. Discussion

Mitochondrial dysfunction has been implicated as one of the disrupted mechanisms for PD (McCormack et al., 2002). The mutations in the PD pathophysiology-related genes such as *Parkin* and *PINK1* have been involved this dysfunction. Pesticides like paraquat or rotenone, playing a role in oxidative stress or inhibiting mitochondrial complex I, respectively, have been used to model PD for decades. In addition, rotenone administration can cause nigral dopaminergic neuron loss and behavioral changes associated with human PD in model organisms (Henchcliffe and Beal, 2008). One strategy to keep dopaminergic neurons alive in the cerebrum is to expose them to growth factors to initiate survival signals such as glial cell-line derived neurotrophic factor (GDNF), neural growth factor, or GH (Hou et al., 1996). Besides inducing cell growth, cell division, and cell regeneration, GH is also a potent stimulator of IGF-I secretion. A clear association between declined IGF-1 by age and PD has been supported by several lines of evidence (Bernhard et al., 2016). Therefore, an increased GH signaling with IGF-1 can be an excellent strategy to inhibit dopaminergic neuron apoptosis during PD.

Apart from oxidative stress and impaired mitochondrial functions, the pathogenesis of PD also involves protein misfolding and aggregation, leading to ER stress. ER stress is a crucial phenomenon in the decision of cell death or survival, since it can trigger autophagy or apoptosis according to the stress level (Corazzari et al., 2017). In the light of these information, our aim in this study was to investigate the protective role of GH following modeling PD in vitro with rotenone treatment in SH-SY5Y and SK-N-AS cells. The forced expression of autocrine GH caused a protective effect on rotenone-induced downregulation of cell viability. Moreover, the rotenone-induced apoptosis was reduced by GH transfection while the autophagy process was triggered in SH-SY5Y cells. These data demonstrated that GH has a neuroprotective role against rotenone treatment through the downregulation of ER stress-related apoptosis in SH-SY5Y neuroblastoma cells.

We used SH-SY5Y and SK-N-AS human neuroblastoma cells, which possess low levels of autocrine GH as consistent with previous study (Zhang et al., 2010). To prove the neuroprotective role of GH in the treatment of PD, SH-SY5Y cells were transfected with GH. The PD model of SH-SY5Y cells was constructed by treating rotenone as previously described (Sherer et al., 2003). Previous studies claimed that rotenone could pass through the blood-brain barrier and induces neuronal dysfunction and ultimately neurodegeneration (Sherer et al., 2003). We found that rotenone reduced the cell viability and growth potential of both neuroblastoma cells, while GH+ cells were less affected by rotenone treatment. The neuroprotective effect of GH was observed starting from 24 h and the effect continued in a time-dependent manner.

Moreover, the anchorage-independent growth of GH+ cells was more evident compared to SH-SY5Y wt cells. The cell survival-promoting role of GH was suggested due to the its relation with IGF-1 which in turn can influence proliferation promoting protein expressions (Sherer et al., 2003). In addition, another important growth factor, VEGF, which has a role in angiogenesis, is related to GH signaling and its proliferation inducing potential.

Dose-dependent rotenone treatment leads cell viability loss, ROS generation, and GRP78 and GADD upregulation in neurons (Corazzari et al., 2017). Rotenone induced unfolded protein response via IRE1α and PERK upregulation in PC12. We demonstrated that rotenone-induced ER stress through the accumulation of CHOP in SH-SY5Y cells, in which GH-overexpressed cells showed lower CHOP levels following rotenone treatment. Various studies also enlighten the effect of rotenone on the induction of ER stress via the upregulation of XBP-1 and activation of CHOP in neuroblastoma cells (Chen et al., 2008). Another study showed that rotenone increased the cleavage of ATF6 and phosphorylation of eIF2α, leading to an increase in apoptosis in neuroblastoma cells (Bernhard et al., 2016). However, studies showed that attenuation of ER stress-based apoptotic process was necessary to reduce the neurotoxicity of therapeutics (Corazzari et al., 2017). Our study observed the levels of CHOP, PERK, p-PERK, p-IRE1α XBP-1, and eIF2α significantly increased following 24-h treatment compared to the early time treatment of rotenone in SH-SY5Y cells. However, rotenone’s 6-h treatment time led to a significant increase of ATF6, XBP-1, and eIF2α levels in SH-SY5Y cells. Forced expression of GH prevented the rotenone-induced expression of ATF6, XBP-1, and eIF2α levels. The downregulation of ATF6 and XBP-1 levels correlated with the loss of CHOP levels in GH+ SH-SY5Y cells.

Interestingly, the expression level of SAPK/JNK was more evident in GH+ cells following rotenone treatment. JNK signaling lead to the stimulation of both autophagy and apoptosis. It was known that the PERK/ATF6/eIF2α pathways have a dual role in cell survival and apoptosis, and ER stress can stimulate the autophagy mechanism through the interaction of IRE1α and mitogen-activated protein kinase-8 (Corazzari et al., 2017). In this study, the attenuated level of ER-stress in GH+ cells was associated with an induction of autophagy after rotenone treatment. The key markers of autophagy; LC3 cleavage, decreased level of p62 were observed in rotenone-treated GH+ SH-SY5Y cells. The expression level of Beclin-1 decreased in a time-dependent manner in SH-SY5Y cells, while there was an adverse effect in GH+ SH-SY5Y cells. Consistent with previous studies, the attenuated-Beclin-1 levels in SH-SY5Y cells were correlated with the induction of apoptosis through interaction with Bcl-2 antiapoptotic members in cancer cells (Lu et al., 2018). However, during the 9-and 24-h treatment of rotenone, the Beclin-1 levels were higher when compared with the control group. These data suggested that GH converted rotenone-induced ER stress to autophagy in SH-SY5Y cells. Previous studies suggested that autophagy plays a dual role in cancer by either suppressing tumor proliferation or inducing resistance to chemotherapeutics. In neuroblastoma tumors, the basal levels of autophagy caused a chemoresistance to hydroxychloroquine (Belounis et al., 2016). In our study, rotenone-induced ER stress was mediated by GH+ to induce autophagy. At this time point, the apoptotic ratio was lower in GH+ cells when compared to SH-SY5Y wt cells.

Moreover, GH led to an increase in the G2/M population in SK-N-AS cells, and rotenone treatment induced the cell cycle arrest at G2/M phase. The apoptosis markers did not produce a consistent response to rotenone treatment in GH+ SK-N-AS cells. SK-N-AS wt cells downregulated caspase-9, caspase-7, and Bcl-2 levels after rotenone treatment. However, rotenone decreased the Bax levels and increased the Bcl-2 levels in GH+ SK-N-AS cells. The neuroprotective GH in apoptotic cell death induced by increasing level of Bcl-2 in SK-N-AS cells. Therefore, GH had a neuroprotective effect on the cell survival of SH-SY5Y and SK-N-AS cells.

In conclusion, GH-mediated ER-stress and autophagy process induced cell viability. Therefore, it can be concluded that GH had a neuroprotective effect by reducing the apoptosis in a mitochondria-dependent manner through the regulation of caspase-7/PARP during rotenone treatment of SH-SY5Y cells (Figure S3). In addition, GH treatment can also eliminate neurotoxicity and neurodegeneration during chemotherapy. Our recent study demonstrated for the first time the forced GH expression prevented the rotenone-induced apoptosis via targeting ER stress and autophagy axis. By this study, we determined the increased autophagy-ER stress induction through related signaling pathways key players’ expression profile. Our research study emphasized the essential role of autophagy and ER stress during rotenone-triggered apoptosis in GH+ SH-SY5Y cells. However, silencing key molecules expressions or rotenone and autophagy inhibitor combined treatments should be performed in order to highlight the molecular machinery underlying the rotenone-induced ER stress-autophagy processes.

## Supplementary

Figure S1 Autocrine GH prevented rotenone-induced cell viability.
SH-SY5Y wt and GH+ cells were treated with 0,5 μM, following drug exposure cells were treated with MitoTracker Red for 15 min. Viable cells were visualized by florescence microscopy. Magnification: 100×.

Figure S2 Rotenone induced ROS generation was inhibited by forced GH expression.
SH-SY5Y wt and GH+ cells were treated with rotenone in time-dependent manner (0–24 h). Following drug exposure, cells were treated with DCFDA dye and trypsinized cells were analyzed by FACS flow cytometer.

Figure S3 The schematic summary of rotenone-induced ER stress-autophagy in autocrine GH SH-SY5Y cells.

## Abbreviation list

ER: Endoplasmic reticulum
GHRP-6: growth hormone-releasing peptide-6
HRP: horseradish peroxidase
GH: human growth hormone
LRRK2: leucine-rich repeat kinase 2
MMP: mitochondrial membrane potential
PD: Parkinson’s disease
PVDF: polyvinylidene difluoride
PI: propidium iodide
ROS: reactive oxygen species
SDS-PAGE: sodium dodecyl sulfate-polyacrylamide gels
UPR: unfolded protein response
