# Supplementary figures and images for: The protective impact of growth hormone against rotenone-induced apoptotic cell death via acting on endoplasmic reticulum stress and autophagy axis
Source: Turk J Biol. 2022 Dec 15;47(1):29–43. doi: 10.55730/1300-0152.2639 (PMC10388008; doi:10.55730/1300-0152.2639)

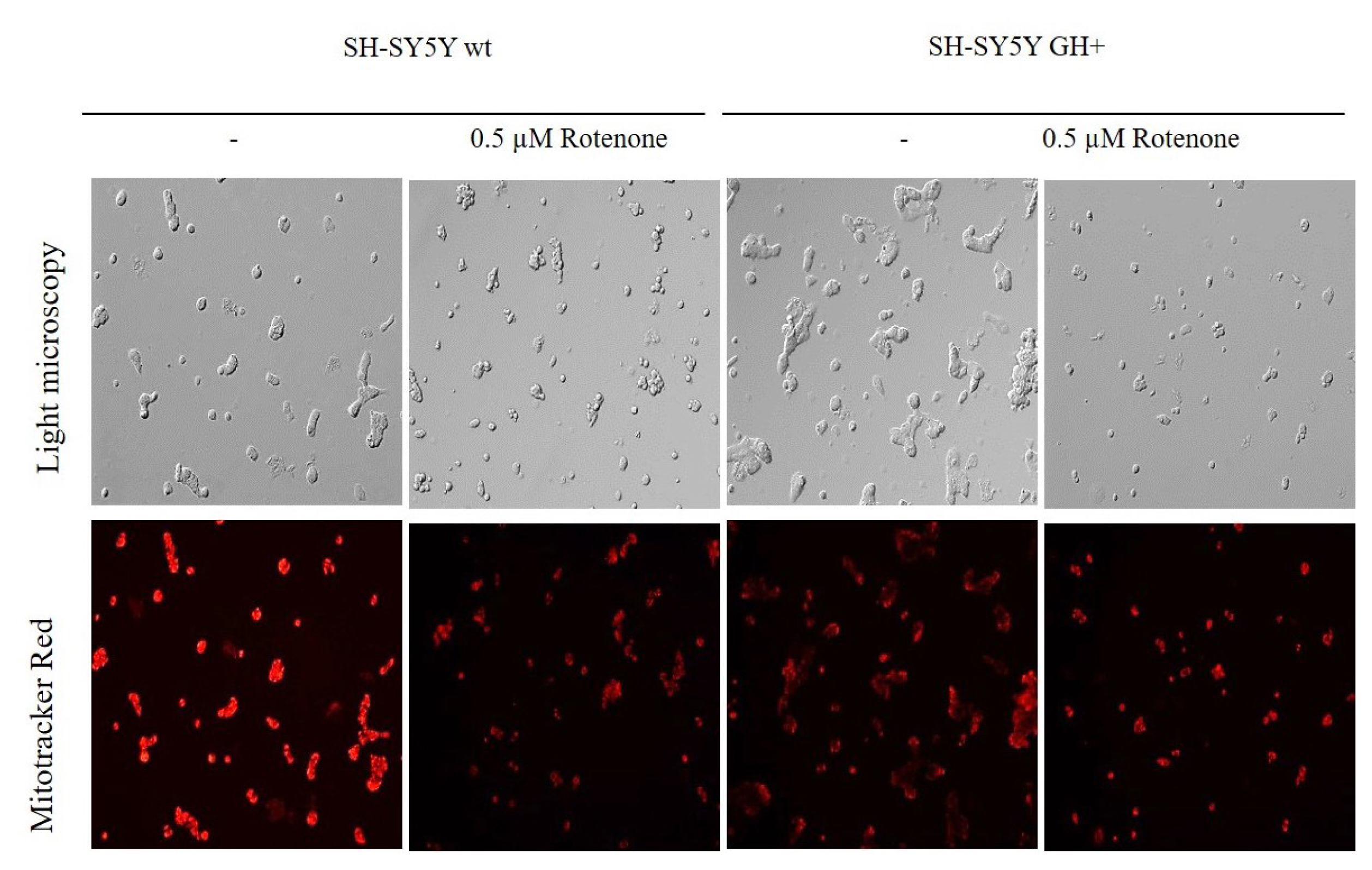

Supplement: Figure S1 — Autocrine GH prevented rotenone-induced cell viability. SH-SY5Y wt and GH+ cells were treated with 0,5 μM, following drug exposure cells were treated with MitoTracker Red for 15 min. Viable cells were visualized by florescence microscopy. Magnification: 100×. [file turkjbiol-47-1-29s1.tif]

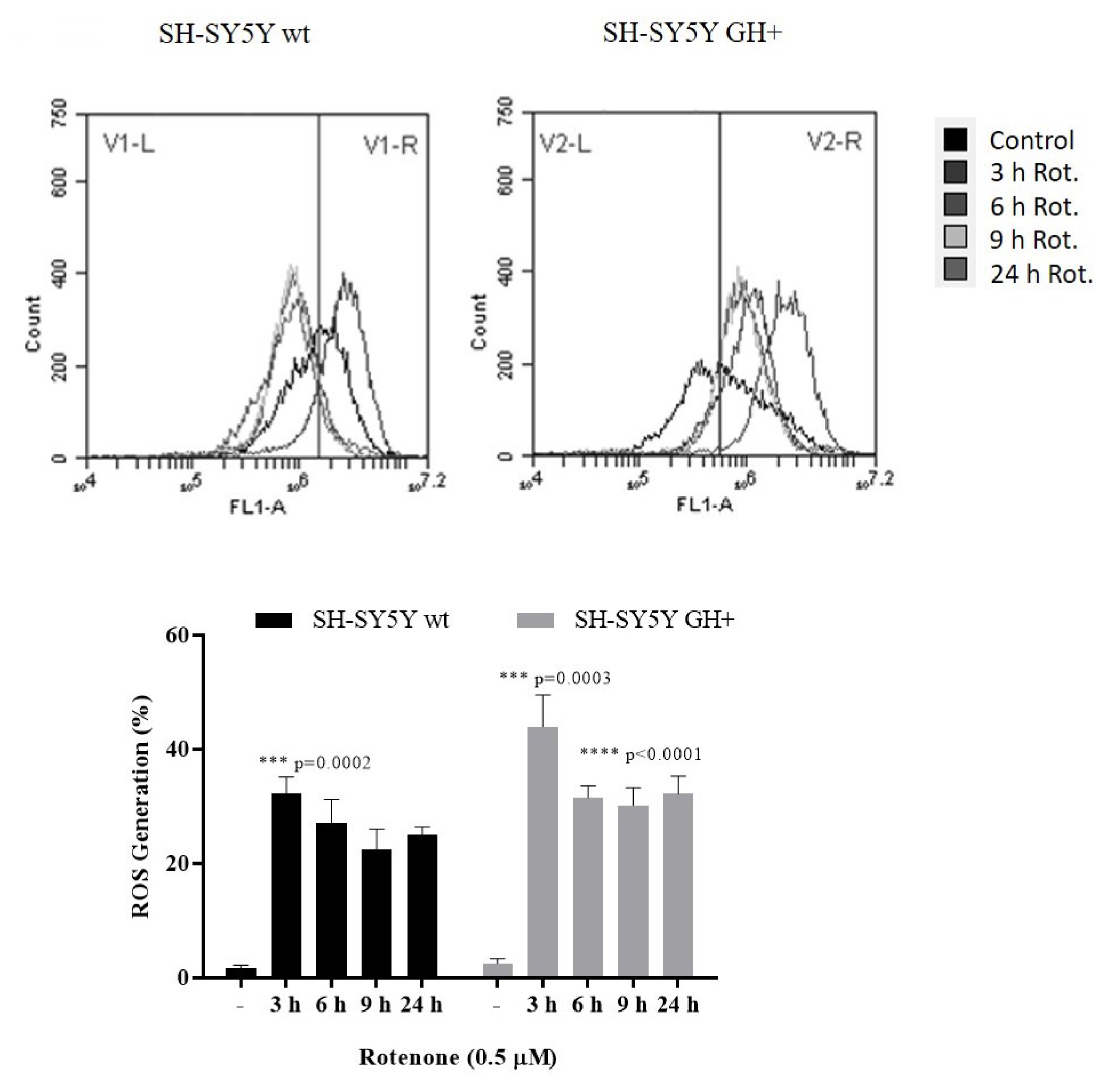

Supplement: Figure S2 — Rotenone induced ROS generation was inhibited by forced GH expression. SH-SY5Y wt and GH+ cells were treated with rotenone in time-dependent manner (0–24 h). Following drug exposure, cells were treated with DCFDA dye and trypsinized cells were analyzed by FACS flow cytometer. [file turkjbiol-47-1-29s2.tif]

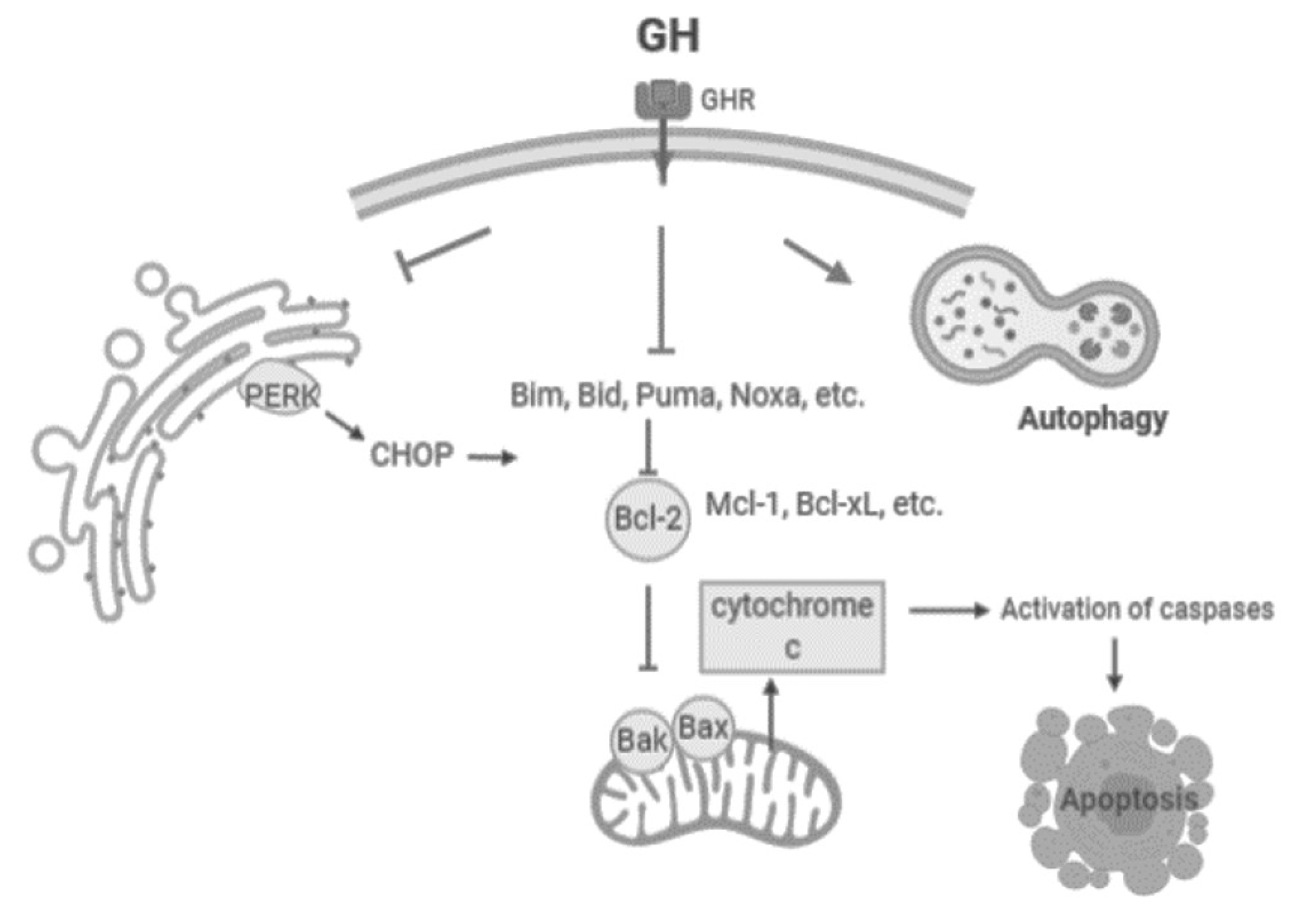

Supplement: Figure S3 — The schematic summary of rotenone-induced ER stress-autophagy in autocrine GH SH-SY5Y cells. [file turkjbiol-47-1-29s3.tif]
